# Human Stem Cell-Derived Models of the Alzheimer’s Disease Neuroimmune System

**DOI:** 10.3390/ijms27167360

**Published:** 2026-08-18

**Authors:** Rose A. Summers, Daphne Quang, Noah R. Johnson

**Affiliations:** 1 Translational Neuroradiology Section, National Institute of Neurological Disorders and Stroke, National Institutes of Health, Bethesda, MD 20892, USA; 2 Cambridge Department of Medicine, University of Cambridge, Cambridge CB2 0AW, UK; 3 University of Colorado Alzheimer’s and Cognition Center, Department of Neurology, University of Colorado Anschutz, Aurora, CO 80045, USA; 4 Linda Crnic Institute for Down Syndrome, University of Colorado Anschutz, Aurora, CO 80045, USA

**Keywords:** Alzheimer’s disease, neuroimmune system, neuroinflammation, human induced pluripotent stem cells, microglia, astrocytes

## Abstract

Mounting evidence implicates dysregulation of the neuroimmune system in Alzheimer’s disease (AD). Neuroimmune cells, namely microglia and astrocytes, have the potential to contribute to AD through mechanisms such as promoting neuroinflammation and propagating amyloid-β (Aβ) and tau aggregates. Human induced pluripotent stem cell (hiPSC)-derived models offer advantages for studying the AD neuroimmune system, such as recapitulating genetic variants associated with the disease and allowing for precise manipulation of human cells in vitro. Here, we provide an overview of modern techniques for generating 2-dimensional (2D) monocultures and co-cultures, 3-dimensional (3D) organoids and assembloids, and chimeras containing hiPSC-derived microglia and astrocytes. Then, we highlight recent studies that have utilized hiPSC-derived neuroimmune models to investigate AD risk variants in genes encoding apolipoprotein E (*APOE*) and triggering receptor on myeloid cells 2 (*TREM2*), mutations known to cause familial AD in genes encoding presenilin 1 (*PSEN1*) and 2 (*PSEN2*) and amyloid precursor protein (*APP*), and trisomy 21 leading to Down syndrome-associated AD (DS-AD). We then briefly summarize recent studies that have utilized hiPSC-derived neuroimmune models lacking disease-associated variants to study the clearance of Aβ and tau aggregates. Finally, we discuss notable limitations of these models and reflect on future directions for this area of research, including the use of cultures with increasing cellular diversity and structural complexity, advancements in live-imaging techniques for detecting AD pathology in vitro, and drug screening.

## 1. Introduction

### 1.1. Alzheimer’s Disease

Alzheimer’s disease (AD) is the most common cause of dementia and a leading cause of death worldwide [1]. A small proportion of AD cases are due to autosomal dominant inheritance of a familial AD genetic mutation (early-onset) or trisomy 21 (Down syndrome), while the majority are sporadic (late-onset) and result from a combination of genetic and environmental factors [1]. The pathophysiological characteristics of AD include the accumulation of amyloid-β (Aβ) and hyperphosphorylated tau aggregates in the brain, paired with progressive neurodegeneration [2]. Notably, Aβ and tau aggregates are present in a range of neurological disorders [3], and AD is a complex disease involving dysregulation of multiple physiological processes, including neuroinflammation [4].

The amyloid precursor protein (APP) is highly expressed in neurons [5] and is cleaved by secretases to yield Aβ peptides that aggregate to form soluble Aβ oligomers (AβOs) and fibrils that then further combine into larger insoluble plaques [6]. AβOs are highly neurotoxic forms of Aβ aggregates due to their propensity to propagate and to interact with cell membranes [6]. Tau is also highly expressed in neurons, and its hyperphosphorylation causes it to aggregate into fibrils, leading to the formation of insoluble neurofibrillary tangles [6]. The specific roles of AβOs, fibrils, plaques, and tau tangles in AD are debated, particularly since drugs targeting Aβ and tau have thus far shown limited and variable success in slowing disease progression and alleviating symptoms [7,8].

Despite the prevalence and societal impact of AD, its etiology is poorly understood, and modern therapies are not curative. Although amyloidosis and tauopathy are undoubtedly involved in AD pathophysiology and have been the primary focus of pre-clinical and clinical research, there is an emerging consensus that the neuroimmune system plays important and targetable roles in the disease. In this introduction, we will summarize the characteristics and functions of the primary neuroimmune cells, studies that implicate neuroimmune involvement in AD, and the advantages of using human induced pluripotent stem cell (hiPSC)-derived models in this area of research. We will then discuss current protocols for generating two-dimensional (2D), three-dimensional (3D), and chimeric hiPSC-derived neuroimmune cultures, followed by the results of recent studies that have utilized these models, with a focus on sporadic and familial AD genetics and on Aβ and tau. Finally, we will reflect on the limitations and future directions of hiPSC-derived models of the AD neuroimmune system.

### 1.2. The Neuroimmune System

The neuroimmune system encompasses the resident innate immune cells of the central nervous system (CNS): namely microglia and astrocytes, though oligodendrocyte progenitor cells (OPCs) have also gained recent attention for their immune functions [9] and brain pericytes also play important roles [10] (Table 1). It is notable that, despite the outdated view that the CNS was an immunologically privileged site protected by a robust blood–brain barrier (BBB), we now know that the peripheral immune system and the CNS are interconnected. The parasagittal dura, a lymphatic structure interfacing with the brain, facilitates the presentation of CNS antigens to T-cells under homeostatic conditions [11]. Furthermore, it is now widely accepted that BBB compromise and related infiltration of peripheral immune cells (e.g., lymphocytes and monocytes) into the CNS occur in numerous disorders, including AD [12,13,14]. However, despite peripheral immune cells communicating with and, in some cases, entering the CNS, neuroimmune cells are distinct and inextricably linked to neurological physiology and pathology [15].

#### 1.2.1. Microglia

Microglia are the primary innate immune cells of the CNS. Although they share functions such as phagocytosis and cytokine secretion with peripheral monocyte-derived macrophages (MDMs), they differ in that they originate from yolk sac-derived hematopoietic progenitor cells (HPCs) that migrate to the neural tube during embryogenesis and remain in the CNS where they proliferate through self-renewal [16]. While the environment is thought to shape microglial identity, supported by studies showing that MDMs can take on a more microglia-like transcriptome upon entering the CNS [17], MDMs are still distinct even after engraftment or infiltration through the compromised BBB [18,19]. Ultimately, multiple factors, including embryonic origin and tissue-specific environment, contribute to microglia exhibiting unique patterns of gene expression. They are the only cells that robustly express cell surface markers transmembrane protein 119 (TMEM119) and purinergic receptor P2RY12 (P2RY12), though they can also be identified by pan-macrophage markers such as ionized calcium-binding adapter molecule 1 (IBA1) [20], and they can exhibit unique morphologies ranging from highly ramified to rod-shaped to ameboid depending on activation and location [21]. Microglia are known to be involved in homeostatic functions, including synaptic pruning [22,23], the clearance of dead and dying cells [24,25], and the release of cytokines and other ligands necessary for initiating and sustaining immune responses and associated regenerative pathways [26]. Several properties of microglia are influenced by factors such as brain region and sex [27,28], and they are highly dynamic cells that survey their environment and rapidly respond to external stimuli. This is partly due to their robust expression of toll-like receptors (TLRs) that can bind various pathogen- or damage-associated molecules and trigger intracellular signaling pathways that initiate and propagate immune responses [29]. When reacting to such stimuli, microglia can take on a diverse range of cellular states, and while it was once common to categorize microglia as “M1” or “M2” when exhibiting characteristics typically associated with inflammation or immunosuppression, respectively, this has been deemed an oversimplification since a vast spectrum of nuanced microglial states exist [30]. Consequently, it is widely accepted that during disease, microglia can contribute to harmful neuroinflammation through mechanisms such as the release of inflammatory cytokines, including tumor necrosis factor (TNF), interferon-γ (IFN-γ), interleukin-6 (IL-6), and interleukin-1β (IL-1β) [31], yet their true involvement in neurological pathology is likely multifaceted.

#### 1.2.2. Astrocytes

Astrocytes perform many immune functions within the CNS. In contrast to microglia that originate from yolk-sac derived macrophage progenitors during development, astrocytes are derived from the neuroectoderm and are mainly replaced through the differentiation of neural progenitor cells (NPCs) in adulthood [32]. Astrocytes express cell type-specific markers including glial fibrillary acidic protein (GFAP), S100 calcium-binding protein β (S100β), and folate enzyme aldehyde dehydrogenase 1 family member L1 (ALDH1L1) [33] and display a characteristic branched morphology with a complex network of thin processes that allow them to directly interface with cells in their vicinity including BBB-associated cells, neurons, and microglia [34]. The morphology and transcriptome of astrocytes can vary depending on factors such as sex [35], age [36], and brain region [36,37]. These cells serve a multitude of homeostatic functions including regulating synapse formation and activity [38], maintaining the BBB [39], supporting oligodendrocytes and consequently maintaining myelin sheaths [40], and working alongside microglia to clear and degrade toxic aggregates [41]. Furthermore, in response to injury, astrocytes can adopt activated phenotypes and release inflammatory cytokines such as IL-1β and IL-6, as well as neurotrophic ligands, to facilitate regenerative processes [42]. Because astrocytes are essential for BBB homeostasis, neuronal health and functionality, and neuroregeneration within the CNS, they are undoubtedly therapeutic targets for neurological pathology. Furthermore, it is important to consider how the neuroimmune functions of astrocytes could be involved in neuroinflammatory diseases such as AD.

#### 1.2.3. Oligodendrocyte Progenitor Cells (OPCs)

Only recently have OPCs been recognized for their roles in the neuroimmune system. OPCs are multipotent cells of neuroectodermal origin that can differentiate into oligodendrocytes in response to normal cell turnover or pathological demyelination, but that also appear to serve homeostatic functions in their immature state [9]. These cells are typically described as bipolar in morphology, but their branching can be more numerous and complex under some circumstances [43]. Markers for OPCs include platelet-derived growth factor receptor-α (PDGFRα), neural/glial antigen 2 (NG2), and SRY-box transcription factor 10 (SOX10) [9,44]. Like other neuroglia, the transcriptomic profile, morphology, and functions of OPCs are highly diverse and dynamic and can be influenced by a variety of factors including brain region, sex, and age [45,46]. Similar to astrocytes, OPCs use their processes to interface with the cells in their vicinity including neurons and BBB-associated cells and, similar to microglia, OPCs phagocytose extracellular waste including myelin debris [9]. Furthermore, OPCs undergo phenotypic changes associated with a range of inflammatory and anti-inflammatory states in response to external stimuli and also secrete cytokines under some conditions [9]. In time, it is expected that the roles these cells play in neuroimmune mechanisms including pathological neuroinflammation will be brought to light.

#### 1.2.4. Other Cell Types

This review will focus on microglia, astrocytes, and, to a lesser extent, OPCs, but it is important to acknowledge that other cell types are inextricably linked with neuroimmunity. One example is pericytes: cells with highly diverse developmental origins, characteristics, and functions that interface with BBB cells and astrocytes, play critical roles in inflammatory processes, and express markers including platelet-derived growth factor receptor-β (PDGFRβ), aminopeptidase N (APN), and NG2 [10]. Additionally, while this review will center on the innate immune mechanisms that may be involved in AD pathophysiology, it is worth noting that all neuroimmune cell types described here communicate with the peripheral adaptive immune system through mechanisms such as antigen presentation to T-cells [47,48,49]. Ultimately, the neuroimmune system is interconnected with, but distinct from, the peripheral immune system and encompasses complex cell types involved in a multitude of homeostatic, regenerative and, in some contexts, pathological processes, many of which are not yet fully understood.

### 1.3. Evidence for Neuroimmune Involvement in Alzheimer’s Disease

Because the neuroimmune system comprises a significant portion of the CNS and is composed of reactive and dynamic cells, alterations in microglia, astrocytes, and OPCs are ubiquitous in neurological injury and disease; however, there is significant evidence to support that neuroimmune dysfunction is not only a consequence of AD but may instead directly contribute to progression. Studies in post-mortem human and mouse brain tissue have established that both microglia and astrocytes are prevalent around Aβ plaques and tau tangles where they exhibit distinct disease-associated phenotypes [50,51]. Indeed, Aβ and tau accumulation correlated with markers of microglial and astrocytic activation in a longitudinal study [52]. In mice, it has also been reported that OPCs cluster near, and extend their processes towards, Aβ plaques [53]. Recent research has provided evidence for specific genetic, cellular, and molecular mechanisms that may underlie the close relationships between neuroimmune cells and AD pathophysiology.

While familial AD is caused by variants of genes that directly contribute to Aβ production such as *APP* and presenilin 1 and 2 (*PSEN1* and *PSEN2*), the strongest genetic risk variant for sporadic AD is the ɛ4 allele of the apolipoprotein E gene (*APOE4*). *APOE4* homozygotes are at a substantially higher risk for developing Aβ and tau pathology as well as related cognitive decline when compared to *APOE4* heterozygotes, and all *APOE4* carriers are at a higher risk compared to those homozygous for the ɛ3 allele (*APOE3*) [54]. Notably, inheritance of the ɛ2 allele (*APOE2*) or the *APOE3* Christchurch variant (*APOECh*) may be protective against AD [55]. *APOE* is expressed in virtually all CNS cell types, but under homeostatic conditions it is most robustly expressed in astrocytes [55]. However, *APOE* expression in microglia is substantially increased in response to disease-associated stimuli including Aβ plaques [56]. The neuroimmune functions of APOE appear to be allele-specific, as one in vitro study of human primary cells showed that *APOE4* microglia and astrocytes adopt more innately inflammatory phenotypes compared to the same cells carrying *APOE3* or *APOE2* [57]. The effects of *APOE* genotype on OPCs have not yet been reported to date, and further investigation would provide valuable information. Relatedly, other neuroimmune-associated gene variants have been identified as risk factors for the onset and progression of sporadic AD, including the triggering receptor expressed on myeloid cells 2 gene (*TREM2*) [58]. Microglia are the primary CNS cell type expressing *TREM2,* which appears to be directly involved in neuroimmune functions including inflammatory cytokine expression [59]. Furthermore, one study has demonstrated that the AD risk variant *TREM2^T96K^* directly impairs the microglial response to Aβ plaques in female mice [60], which may be relevant to AD as a sexually dimorphic disease that occurs predominantly in women [61]. These findings, among many others, provide ample evidence that prominent risk variants for sporadic AD act, at least in part, through their effects on neuroimmune cells.

Genetics aside, characteristic AD pathophysiology is necessarily linked to the neuroimmune system because neuroimmune cell types are responsible for clearing neurotoxic waste from the CNS to maintain homeostasis. Microglia are directly involved in the brain’s response to extracellular and intracellular protein aggregates by facilitating their removal and supporting consequent neuroregenerative pathways [62,63]. Mounting evidence indicates that astrocytes can also serve these functions [41], though in some cases they appear to act in concert with microglia to phagocytose molecules such as Aβ [64]. Additionally, OPCs have phagocytic capabilities, primarily in clearing myelin debris though they can also take up diverse substrates including Aβ [65]. It has long been thought that microglia are primarily responsible for clearing protein aggregates from the CNS, yet dysfunctional microglia may also propagate Aβ and tau aggregates throughout the brain [66,67,68], and this could also be true of other neuroimmune cell types.

On a molecular scale, dysregulation of several distinct but connected neuroimmune pathways can be associated with AD. The family of nuclear factor-κ light chain enhancer of activated B cells (NFκB) transcription factors are known to play essential roles in both peripheral immune and neuroimmune functions through involvement in responses to inflammatory stimuli, including TLR-mediated pathways [69]. Transcriptomic analyses of post-mortem human brain tissue have identified CNS cells from AD patients with enrichment of genes associated with TLR4 activation and NFκB pathways [70,71]. However, the potential role of NFκB signaling in AD appears complex in that depleting this pathway in astrocytes can worsen Aβ pathology in mice [70] while, conversely, depleting it in microglia can inhibit tau propagation and improve cognition [68]. Other molecular neuroimmune mechanisms implicated in AD include mitogen activated protein kinase (MAPK) signaling pathways; more specifically, genes associated with p38 MAPK are enriched in post-mortem brain tissue from patients with AD and a mouse model of AD [72]. While p38 MAPK is expressed by neurons, it is also highly expressed by glia and one study found that microglial expression of p38 MAPK increased with age in a mouse model of tauopathy and was associated with an inflammatory phenotype [73]. Furthermore, both microglia and astrocytes increase p38 MAPK signaling in response to Aβ and tau [73,74], while inhibiting p38 MAPK was shown to improve the phagocytosis of tau aggregates by primary murine microglia [75] and reduce the reactivity of primary murine astrocytes to Aβ [74]. The Janus kinase/signal transducer and activator of transcription (JAK/STAT) pathway is known to be involved in regulating interferon signaling in neuroimmune cells, and mounting evidence supports that this pathway is altered in AD [76]. NFκΒ, MAPK, and JAK/STAT signaling are several interconnected pathways that can function agonistically in neuroinflammation and appear to be involved in AD pathophysiology, particularly in the microglial and astrocytic responses to amyloidosis and tauopathy, yet it remains unknown how, and to what extent, dysregulation of these pathways may contribute to the disease. Furthermore, these inflammatory mechanisms are necessary for maintaining homeostasis and are only pathological in specific contexts of dysregulation, and the same pathway may be either beneficial or detrimental in the context of certain cell types and stimuli.

In sum, the genetic risk variants, the cell types associated with Aβ and tauopathy, and the molecular pathways enriched in the brains of patients with AD consistently point to neuroimmune dysregulation. Since it is increasingly clear that neuroimmune mechanisms will be therapeutic targets for AD, a new question emerges: which models are best suited for this area of research?

### 1.4. The Need for hiPSC-Derived Models

Genetically modified mice can develop some aspects of AD pathophysiology, including Aβ aggregation and tauopathy, and can model human risk variants such as *TREM2^T96K^* and *APOE4* [77], but no animal model fully recapitulates all aspects of human disease. While murine models have been valuable in pre-clinical research and drug testing, many results from these models do not translate clinically due to factors including intrinsic biological differences between species [78]. For example, humans possess larger and more complex astrocytes with distinct responses to certain stimuli [79,80], microglia with unique patterns of gene expression [81], subtype heterogeneity, and phagocytic capacity [82], and OPCs with distinct metabolism when compared to murine cells [83]. Furthermore, meta-analyses of differential gene expression between human and mouse neuroimmune cells, including cells treated with the inflammatory stimulus lipopolysaccharide (LPS), which activates TLR4-dependent inflammation [84], have revealed that some patterns of gene expression are not conserved across species, and some even appear contradictory between humans and mice [85]. While it is challenging to know the extent and significance of such differences, especially because few studies have directly compared the transcriptome and function of human and mouse neuroimmune cells, it is clear that murine models alone are insufficient for facilitating translatable AD research. Non-human primates may offer some advantages as they are evolutionarily closer to humans, yet they come with complex care requirements and ethical considerations and do not naturally develop AD [86]; the value in using human models is evident.

The advent of methods for reprogramming adult somatic cells into hiPSCs [87], and the consequent development of protocols for differentiating hiPSCs into a range of mature cell types, co-cultures, and 3D structures, have created new opportunities for studying the human neuroimmune system [88,89]. For some research questions, hiPSC-derived in vitro models are uniquely advantageous for their simplicity: uncomplicated by multi-organ systems and multi-cell crosstalk, they can be precisely manipulated. When these complexities are important, hiPSC-derived cells and organoids can also be transplanted into animals to generate chimeras, creating the valuable opportunity to study human cells in vivo [89,90].

## 2. hiPSC-Derived Models of the Neuroimmune System

### 2.1. 2D Monocultures and Co-Cultures

Numerous protocols for differentiating monocultures of cells that are transcriptomically, morphologically, and functionally similar to those of the human neuroimmune system have been published. Supplementing cell culture media with ligands to facilitate hiPSC-derived microglia (iMG) differentiation can yield cells with characteristic microglial gene expression, morphology, phagocytic ability, and responsiveness to stimuli [91,92,93,94]. This process generally occurs in stages over several weeks, first generating hematopoietic progenitor cells (HPCs), then immature, and then mature iMG [91,92,93,94]. However, there has been a recent shift towards protocols that combine expression of transcription factors with minimal media supplementation, which can shorten this differentiation to just 8 days [95]. Furthermore, one 2025 study reported that inducing expression of 6 transcription factors with no media supplementation can yield iMG in just 4 days [96]. Similarly, differentiation of hiPSC-derived astrocytes (iAS) can be accomplished through multiple supplementation-based approaches, yielding cells with characteristic astrocytic gene expression, morphology, and response to stimuli [97,98,99]. Transcription factor expression combined with defined media supplementation can accelerate iAS differentiation and control the astrocytic subtypes generated [100]. Protocols have also been described to generate hiPSC-derived OPCs (iOPCs) through defined media supplementation [101], and inducing expression of transcription factors such as SOX10 reportedly increases the efficiency of deriving iOPCs from NPCs [44]. However, because iOPCs are commonly generated as intermediates for mature oligodendrocytes, their neuroimmune functions have not yet been characterized or compared to those of their in vivo counterparts.

While hiPSC-derived monocultures are valuable tools for studying some cell-type-specific mechanisms, they fail to recreate the complex multi-cell crosstalk present in vivo; co-cultures are advantageous in this aspect. Given that microglia and astrocytes are known to physically interface and communicate through bidirectional paracrine signaling [102], co-culture systems including both iMG and iAS have clear value. Such co-cultures are especially relevant in the context of studying AD because it has been shown that iMG and iAS together clear Aβ more efficiently than either cell type individually [64]. Furthermore, co-culture systems including iMG, iAS, and hiPSC-derived neurons (iN) have been developed to more comprehensively mimic the cellular composition of the CNS [103,104].

### 2.2. 3D Organoids and Assembloids

Compared with 2D models, 3D hiPSC-derived systems share more similarities with the physical structure of human CNS tissue and can better support complex cellular morphology and cell–cell connections [89]. Traditional neural organoids predominantly contain cell types derived from the neuroectoderm (e.g., NPCs, neurons, and astrocytes) [105], but protocols have evolved over time to allow for the integration of mesodermal-derived microglia to grant immune competence. The presence of these iMG can improve neuronal functionality [106] as well as long-term cell viability in these models [107]. These microglia-containing organoids (MCOs) can be generated either through co-culturing mature iMGs with mature neural organoids or through co-culturing HPCs and NPCs that develop together and give rise to MCOs [108]. While traditional MCO differentiation approaches relied on media supplementation alone, it has been more recently shown that inducing expression of the transcription factor PU.1 in a subset of hiPSCs within neural organoids, in combination with media supplementation, can be used to create a highly controllable ratio of iMG [109]. Within the last three years, significant advancements have been made in the integration of OPCs and myelinating oligodendrocytes within neural organoids through media supplementation alone [110] or in combination with inducible expression of transcription factors such as SOX10 [111], and these myelinating organoids can be co-cultured with microglia to generate models of human tissue that include most of the key neuroimmune cell types (i.e., microglia, astrocytes, and OPCs) [110,111].

Multiple organoids can be combined together into assembloids to model connections between cells in different regions of the CNS [112]. In the study of the AD neuroimmune system, one highly relevant use of assembloids comes from the fusion of neural organoids modeling different brain regions to enable the study of region-specific microglial phenotypes [113]. Similar models can be useful for understanding how known regional diversity of neuroimmune cells, not only iMG but also iAS and iOPCs, contribute to the distinct regional vulnerability of the human brain to AD neuropathology [114].

### 2.3. Chimeras

All hiPSC-derived models are limited by how cellular identity and function are constantly shaped by the cellular microenvironment, yet no modern in vitro culture system can fully replicate the conditions present in vivo. To overcome this, hiPSC-derived cells and organoids can be transplanted into the brains of immunodeficient animals. For example, human HPCs or mature iMG can be transplanted into the mouse brain, where they mature into cells with characteristic microglial gene expression, complex ramified morphologies, and phagocytic functions [115,116]. Similarly, co-transplantation of human HPCs and NPCs gives rise to a range of human CNS cell types, including microglia, astrocytes, and neurons, within the murine brain [117]. As with in vitro cultures, iOPCs can be integrated into chimeric models [117], but their neuroimmune functions in this context have not yet been thoroughly studied. Although these in vivo transplantation models require significant resources and expertise, it is also possible to section murine brains and combine the slice cultures with hiPSC-derived cells, including iMG or whole MCOs [118,119]. Following co-culture with mouse brain tissue in vitro or in vivo, hiPSC-derived neuroimmune cells can adopt complex morphologies and gene expression signatures characteristic of the human brain, including those associated with astrocyte-microglia communication [115,116,117,118,119].

Despite the benefits of combining hiPSC-derived neuroimmune cultures with animals to create a chimera, there are also notable drawbacks. Because animals must be immunocompromised to facilitate xenotransplantation, the loss of peripheral immune cells can have widespread effects on development and physiology. Furthermore, because transplanted cells in chimera engage in constant crosstalk with animal cells, it may dampen some human-specific aspects of the hiPSC-derived models. Finally, creating and maintaining chimeras also requires significant ethical considerations [120]. Ultimately, hiPSC-derived 2D, 3D, and chimeric neuroimmune models each have distinct weaknesses and are also uniquely well-suited to answer specific research questions (Table 2).

## 3. hiPSC-Derived Neuroimmune Models of AD-Associated Genetics

Because hiPSCs can be reprogrammed from the somatic cells of AD patients or healthy subjects, and can be genetically edited, hiPSC-derived neuroimmune models are well suited for investigating genetic contributions to both sporadic and familial AD.

### 3.1. Sporadic AD Risk Variants

#### 3.1.1. APOE

Findings from post-mortem human brain tissue, human neuroimaging, and murine models indicate that *APOE4* is associated with inflammatory neuroimmune cell phenotypes and enhanced amyloidosis and tauopathy [121,122,123,124], but recent hiPSC-based experiments have provided new insights into specific cellular and molecular mechanisms underlying these observations.

*APOE4* iMG in monocultures and in MCOs exhibited lysosomal dysfunction, increased lipid droplet accumulation, and impaired pinocytosis (a process important for clearing soluble forms of Aβ) when compared with *APOE3* iMG [125,126]. Conversely, iMG carrying the protective *APOECh* had decreased lipid droplet accumulation in response to Aβ in monocultures, and reduced tau in *PSEN1* mutant iN co-cultures and MCOs relative to *APOE3* iMG [127]. It is clear that *APOE* genotype intrinsically influences properties of microglia involved in the clearance of toxic proteins and lipid droplet accumulation, which, in turn, affect their inflammatory responses. More specifically, triglyceride synthesis and lipid droplet formation were necessary for NF-κB signaling in iMG exposed to LPS, and inhibiting triglyceride synthesis in *APOE4* iMG reduced inflammatory gene expression more potently than in *APOE3* iMG [128]. Relatedly, *APOE4* iMG showed increased lipid droplet accumulation after exposure to Aβ fibrils in comparison to *APOE3* cells, and higher lipid droplet accumulation correlated with expression of genes associated with NFκB signaling (e.g., *TNF*) [129], further highlighting the role of *APOE* variants in the neuroimmune response to amyloidosis. Moreover, *APOE4* iAS have shown increased responsiveness to inflammatory stimuli such as IL-1β and impaired lipid metabolism and lysosomal function compared to *APOE3* iAS [130,131]. While the role of APOE as a lipid transporter has long been known, these studies in hiPSC-derived models highlight how the AD risk variant *APOE4* perturbs lipid metabolism in neuroimmune cells and contributes to inherently inflammatory and reactive glial phenotypes. Furthermore, the protective *APOE2* and *APOECh* variants result in anti-inflammatory and non-reactive glial phenotypes. Although analyses of post-mortem human brain tissue have revealed differing transcriptomic signatures of *APOE3* OPCs relative to *APOE2* OPCs [132], the effects of APOE genotype on the neuroimmune functions of iOPCs have yet to be investigated.

#### 3.1.2. TREM2

Similar to *APOE4*, the AD risk variant *TREM2^R47H^* resulted in an intrinsically inflammatory iMG phenotype along with heightened responsiveness to LPS and IFNy and impaired phagocytosis of Aβ in monocultures [133]. When these iMG were transplanted into the mouse brain to form chimeric models, they produced synapse loss [133]—an event known to drive neurodegeneration in AD [134]. As it is thought that AD-associated *TREM2* variants primarily exert loss-of-function pathological effects, it is relevant to note that increasing TREM2 activity with an activating antibody in iMG monocultures enhanced lipid catabolism and reduced lipid droplet accumulation following treatment with myelin debris [135], as well as increasing homeostatic functions such as phagocytosis in murine microglia [135], demonstrating that this pathway may be therapeutically targetable.

Furthermore, it will be valuable for future studies to utilize male and female hiPSCs to investigate how biological sex, either alone or in combination with AD risk variants, may contribute to neuroimmune cell dysfunction, as this topic remains poorly understood despite the increased prevalence of AD in women, sexual dimorphisms observed in animal models of AD, and sex-specific characteristics of neuroimmune cells [136]. It is also necessary to acknowledge that there are many genetic risk variants linked to AD that have not been discussed here, but that could be studied in hiPSC-derived neuroimmune models. For example, variants in the gene *ABCA7* have been linked to heightened sporadic AD risk specifically in African Americans, a group disproportionally affected by the disease but historically understudied in this context, with some evidence indicating that these variants could contribute directly to neuroimmune dysregulation [137].

### 3.2. Familial AD

Recent studies utilizing hiPSC-derived models indicate that familial AD variants in *PSEN1*, *PSEN2*, or *APP* not only influence Aβ peptide formation but can also directly influence neuroimmune function.

#### 3.2.1. PSEN1 and PSEN2

A *PSEN2* mutation associated with familial AD in iMG and iAS elicited morphology and gene expression associated with an inflammatory phenotype, as well as exaggerated inflammatory cytokine generation in response to Aβ [138]. Relatedly, several different *PSEN1* mutations yield increased expression of genes associated with inflammation (e.g., *TNF*) in iAS monocultures, even in the absence of inflammatory stimuli [139]. *PSEN1* mutations further disrupt iAS homeostasis through mechanisms such as altered glucose metabolism [140], which is essential for supporting neuronal homeostasis, and disruption could contribute to neurodegeneration and AD [141]. While *PSEN1* and *PSEN2* variants likely contribute to familial AD pathogenesis through multiple synergistic mechanisms occurring across a range of cell types, such studies highlight the importance of microglial and astrocytic neuroimmune dysfunction. Similar to risk variants of *TREM2* and *APOE,* it appears that these mutations can drive innately inflammatory and reactive neuroimmune phenotypes.

#### 3.2.2. Down Syndrome and APP

Down syndrome (DS) results from the triplication of chromosome 21, which contains the *APP* gene, and leads to the development of AD pathology including amyloid, tauopathy, neurodegeneration, and related dementia in the majority of adults [142]. DS iMG in MCOs exhibited elevated synaptic pruning, and in chimeric mice exhibited increased interferon signaling when compared to disomic control cells [143]. DS iAS displayed increased expression of genes associated with neuroimmune function, among other alterations, when compared to disomic controls in one study [144]. Notably, DS is a complex developmental disorder that imparts dysregulation across all organ systems and cell types and is distinct from other forms of familial and sporadic AD; however, neuroimmune dysfunction is a clear contributor to DS-associated AD pathology that is also relevant to the development of novel treatments [145]. For example, silencing one copy of chromosome 21 with x-inactive specific transcript (Xist) RNA has been previously validated in DS iAS [146].

While the Swedish *APP* variant (*APPSwe*), known to cause familial AD, did not significantly alter the inflammatory phenotype or phagocytic capacity of iMG in one study [147], it reportedly increased the responsiveness of hiPSC-derived pericytes exposed to TNF and IL-1β in another [148]. Future studies will further elucidate the role of this and other *APP* variants in perturbing neuroimmune function in different cell types.

Taken together, the studies discussed throughout this section reveal an overarching trend of variants associated with both familial and sporadic AD predisposing hiPSC-derived neuroimmune cells to innately inflammatory phenotypes with heightened responsiveness to inflammatory stimuli. However, each variant uniquely affects specific cell types and molecular pathways, and further research is needed to clarify how these neuroimmune alterations contribute to disease in patients.

## 4. hiPSC-Derived Neuroimmune Models of AD-Associated Pathophysiology

### 4.1. Amyloid-β

iMG within MCO have been shown to phagocytose Aβ and protect iN and iAS from pathology associated with chronic Aβ exposure [149], consistent with the view that the neuroimmune system is essential for the clearance of aggregates. Interestingly, chronic Aβ treatment was insufficient to elicit inflammatory cytokine signaling, such as TNF and IL-1β, from these cells, although they were responsive to LPS [149]. Relatedly, it has been reported that iAS change morphology but do not initiate NF-κB signaling in response to Aβ exposure [150]. These findings support the premise that microglial and astrocytic inflammation in AD is not merely a response to Aβ but rather is a result of amyloidosis in combination with tauopathy and cumulative genetic and environmental factors. However, other studies provide evidence for Aβ exposure alone driving neuroimmune cell dysfunction. For example, iMG showed impaired function of the mechanosensitive ion channel PIEZO1 after exposure to Aβ, with PIEZO1 being involved in the recognition and clearance of amyloid plaques [151]. Likewise, iAS exposed to Aβ had intracellular Aβ accumulation and related metabolic dysfunction [152]. Counterintuitively, neuroimmune cells are necessary for the clearance of Aβ and prevention of related neurodegeneration, yet Aβ seems to directly impair the mechanisms necessary for this clearance; perhaps hiPSC-derived models can further explore this relationship and identify methods of improving Aβ clearance by both microglia and astrocytes in AD.

### 4.2. Τau

Unlike Aβ aggregates, tau tangles are predominantly intracellular [2]; however, tau within dead neurons can be phagocytosed by microglia and astrocytes, and tau aggregates are also present extracellularly in the AD brain [153]. Of relevance, extracellular tau monomers and fibrils were taken up by iMG in monocultures, with fibrils being more difficult for the cells to degrade [154], possibly reflecting how neurofibrillary tangles are particularly challenging for the neuroimmune system to clear in the AD brain. iAS also took up extracellular tau fibrils in monocultures, and tau isolated from post-mortem AD brain tissue elicited a stronger inflammatory response from iAS when compared to tau isolated from healthy control brains [155]. This may relate to how tau in the AD brain is distinctly prone to taking on pathological conformations, including hyperphosphorylated neurofibrillary tangles, and some astrocytic surface receptors linked to inflammatory pathways can differentiate between tau conformations, including monomers and fibrils [156]. In light of recent reports that astrocytes and microglia may also propagate tau aggregates, it is relevant to note that iAS can internalize tau fibrils, fail to degrade them, and then transfer them to healthy neighboring iAS and iN in co-cultures [155,157]. This transfer, generally involving mechanisms including tunneling nanotubes [158] and exosomes [159], protects tau from degradation from the extracellular environment and may be critical for the development of tauopathy in AD.

Clearly, hiPSC-derived culture systems will continue to be useful in revealing specific processes through which neuroimmune cells respond to, internalize, degrade, and propagate both Aβ and tau in AD. Importantly, multiple genetic variants discussed in the previous section are known to directly influence these functions. For example, *APOE4*-driven lipid droplet accumulation in iMG may impair cellular mechanisms necessary for Aβ uptake and degradation [129], *APOEch* iAS cleared intraneuronal tau from iN co-cultures more efficiently than *APOE3* iAS [160], and conditioned media specifically from lipid droplet-laden iMG induced phosphorylated tau pathology in iN [129]. Furthermore, loss-of-function TREM2 mutations are known to impair phagocytosis [161], explaining why *TREM2^R47H^* contributed to impaired phagocytosis of Aβ by iMG [133], and may similarly impair tau clearance—though the results from some studies on this topic are conflicting [58]. Ultimately, it will be important for future research to address how different conformations of tau (e.g., monomers, hyperphosphorylated fibrils, and tangles) and Aβ (e.g., peptides, fibrils, AβOs, and plaques), in combination with genetic variants linked to AD, affect these processes.

## 5. Discussion

### 5.1. Limitations

All hiPSC-derived models, including those of the AD neuroimmune system, have notable limitations. For one, epigenetic aberrations that impair pluripotency can result from incomplete reprogramming of adult somatic cells into hiPSCs or can be acquired later in culture. These aberrations can be minimized through strategies such as optimized reprogramming techniques but may still contribute to hiPSC line heterogeneity. Reprogramming also leads to partial removal of the epigenetics that contribute to age-related gene expression, resulting in hiPSC-derived cells that may be developmentally immature in comparison to adult primary cells. However, one study has found that while age-related transcriptomic signatures were largely lost during the reprogramming of fibroblasts to hiPSCs, iNs differentiated from these cells exhibited distinct transcriptional signatures when from donors under or over the age of 40, suggesting that hiPSC-derived cells may retain some age-associated characteristics. Still, these cells may be inherently limited in their ability to model age-related disorders such as late-onset AD. Furthermore, hiPSC-derived models are typically cultured for weeks to months, far shorter than the years-to-decades over which AD neuropathology and neurodegeneration occur. To highlight how impactful culture times can be in this context, one publication reported that iN only switched from fetal-like to adult-like tau splicing after a full year in culture [162]. Another barrier to studying aging in 3D hiPSC-derived models specifically comes from how most neural organoids lack vasculature and so nutrients cannot penetrate deep within the tissue, resulting in necrotic cores that expand over time.

Integration into complex hiPSC-derived models such as chimeras, rather than monocultures, can improve maturity of neuroimmune cells, including microglia [163], and recent protocols for culturing organoids as air-liquid interface slice cultures [164] or creating organoids with functional vasculature [165] may improve long-term viability. Additionally, controlled expression of genes known to contribute to premature aging, including progerin, or treatment with compounds that promote cell stress, including Fludarabine, have altered age-related characteristics of hiPSC-derived neurons [166,167], highlighting the feasibility of recreating aspects of cellular aging without the need for prolonged culture times. However, further research characterizing how age-related transcriptomic and molecular signatures change throughout reprogramming and differentiation, and how cells age within hiPSC-derived models in comparison to the human brain is needed. Additionally, comprehensive transcriptomic and functional validation of hiPSC-derived cells remains important as protocols for developing these models are still evolving, and variability in the genetic background, reprogramming, and culture conditions of hiPSCs can contribute to significant heterogeneity [168].

### 5.2. Future Directions

While many 2D, 3D, and chimeric hiPSC-derived models have thus far shown promise for modeling aspects of the AD neuroimmune system, it will be valuable for future studies to take advantage of the increasingly complex culture systems that are being developed, including vascularized, myelinating, and region-specific organoids and assembloids [169]. Moreover, while microglia and astrocytes are the primary neuroimmune cells of the CNS and have established involvement in AD, it has become increasingly clear that hiPSC-derived models containing other neuroimmune cell types, such as MCOs that include iOPCs and pericytes [170], should be further studied in this context.

In addition to the continued development of in vitro systems, a key priority for future studies will be the integration of longitudinal live-imaging technologies with hiPSC-derived models [171], such as MCOs, to enable real-time tracking of pathological processes in AD, including Aβ aggregation, tau propagation, and cell type-specific responses. The incorporation of refined fluorescent probes with high specificity for pathological protein conformations, combined with high-content imaging platforms, will support repeated, non-destructive measurements within intact 3D cultures. This approach has the potential to improve drug screening by enabling longitudinal assessment of therapeutic efficacy while simultaneously monitoring cellular viability and toxicity.

Another important direction in AD research is the expanded use of hiPSC-derived neuroimmune models for drug discovery. By combining human genetic backgrounds with multicellular complexity, these systems will enable the evaluation of therapeutic candidates in a physiologically relevant context [172]. MCOs can capture both cell type-specific responses and emergent neuroimmune interactions, such as inflammation, phagocytosis, and protein clearance. Pairing these models with live imaging will enable real-time assessment of treatment effects on dynamic disease processes while distinguishing therapeutic benefit from off-target toxicity.

## 6. Conclusions

There is an emerging consensus that the neuroimmune system plays an integral role in AD pathophysiology. However, further research is needed to elucidate the specific involvement of different neuroimmune cells (e.g., microglia, astrocytes, and OPCs) in disease onset and progression. Modern protocols have been adapted and optimized to facilitate differentiation of hiPSCs into diverse neuroimmune models well-suited to investigate this topic. The recent publications discussed throughout this review demonstrate how these models may be utilized for studying how the human genetics underlying familial and sporadic AD can directly perturb neuroimmune processes as well as how neuroimmune cells interact with the hallmarks of AD pathology: Aβ and tau aggregates. Because these models have inherent limitations, including an inability to recapitulate the cell-type diversity, 3D tissue structures, and aging of in vivo physiology, they require extensive validation and should be used to complement in vivo and clinical research. Nonetheless, as hiPSC-derived neuroimmune models are becoming more controllable, biologically relevant, and specialized, they will provide valuable insights into the etiology and pathogenesis of AD and inform advancements in patient care through the identification of therapeutic targets and drug screening.

## Figures and Tables

**Table 1:** Primary cell types involved in the neuroimmune system.

Cell Type	Cell Markers	Main Known Functions	Refs.
Microglia	IBA1, P2RY12, TMEM119	Phagocytosis, cytokine secretion, synaptic pruning, toxic protein clearance	[15,16,17,18,19,20,21,22,23,24,25,26,27,28,29,30,31]
Astrocytes	GFAP, S100β, ALDH1L1	Neuroinflammatory signaling, BBB maintenance, toxic protein clearance, neuroregeneration	[32,33,34,35,36,37,38,39,40,41,42]
OPCs	PDGFRα, SOX10, NG2	Phagocytosis, myelin maintenance, remyelination, neuroimmune modulation	[9,43,44,45,46]
Pericytes	PDGFRβ, APN, NG2	BBB maintenance, neuroinflammation, regulation of neurovascular coupling	[10]

**Table 2:** Advantages and disadvantages of hiPSC-derived models of the neuroimmune system.

Model Type	Advantages	Disadvantages	Refs.
2D monocultures	• Fast development • Relatively low cost • Useful for studying single human cell types in isolation	• Lack multi-cell type crosstalk • Limited biological relevance due to differences in structure and cell type diversity from in vivo systems	[95,96,97,98,99,100,101,102,103,104,105,106]
2D co-cultures	• Fast development • Relatively low cost • Useful for studying simple crosstalk between multiple human cell types	• Limited biological relevance due to differences in structure from in vivo systems	[66,107,108,109]
3D organoids	• Tissue-like structure supports complex human cell morphologies and phenotypes • Useful for studying complex crosstalk between multiple human cell types	• Slow development • High cost • Lack of multi-region crosstalk • Limited biological relevance due to differences in complex tissue structure and regionality from in vivo systems	[93,110,111,112,113,114,115,116]
3D assembloids	• Tissue-like structure supports complex human cell morphologies and phenotypes • Useful for studying complex crosstalk between multiple human cell types and brain regions	• Slow development • High cost • Limited biological relevance due to differences in complex tissue structure from in vivo systems	[117,118]
Chimeras	• In vivo (xenotransplant) or ex vivo (slice culture) tissue supports complex human cell morphologies and phenotypes • Useful for studying single or multiple human cell types within complex living tissue	• High cost • Ethical considerations • Significant expertise required • Limited biological relevance due to immunocompromised animals and crosstalk between cells of different species	[120,121,122,123,124,125]

## Data Availability

No new data were created or analyzed in this study. Data sharing is not applicable to this article.

## Abbreviations

The following abbreviations are used in this manuscript:

2D	Two-dimensional
3D	Three-dimensional
Aβ	Amyloid-β
AβO	Amyloid-β oligomer
AD	Alzheimer’s disease
ALDH1L1	Folate enzyme aldehyde dehydrogenase 1 family L1
APN	Aminopeptidase N
APOE	Apolipoprotein E
APOE2	Apolipoprotein ε2
APOE3	Apolipoprotein ε3
APOE4	Apolipoprotein ε4
APOEch	Apolipoprotein ε3 Christchurch variant
APP	Amyloid precursor protein
APPswe	Amyloid precursor protein Swedish variant
BBB	Blood–brain barrier
CNS	Central nervous system
DS	Down syndrome
DS-AD	Down syndrome-associated Alzheimer’s disease
GFAP	Glial fibrillary acidic protein
hiPSC	Human induced pluripotent stem cell
HPCs	Hematopoietic progenitor cells
iAS	Human induced pluripotent stem cell-derived astrocytes
IFN-γ	Interferon-γ
iHPCs	Human induced pluripotent stem cell-derived hematopoietic progenitor cells
IL-6	Interleukin-6
IL-1β	Ιnterleukin-1β
iMG	Human induced pluripotent stem cell-derived microglia
MCOs	Microglia-containing organoids
iN	Human induced pluripotent stem cell-derived neuron
iNPCs	Human induced pluripotent stem cell-derived neural progenitor cells
iOPCs	Human induced pluripotent stem cell-derived oligodendrocyte progenitor cells
JAK/STAT	Janus kinase/signal transducer and activator of transcription
LPS	Lipopolysaccharide
MAPK	Mitogen-activated protein kinase
MDM	Monocyte-derived macrophages
NFκB	Nuclear factor κ-light chain enhancer of activated B cells
NPCs	Neural progenitor cells
OPCs	Oligodendrocyte progenitor cells
P2RY12	Purinergic receptor P2RY12
PDGFRα	Platelet-derived growth factor receptor-α
PSEN1	Presenilin 1
PSEN2	Presenilin 2
TF	Transcription factor
TLR	Toll-like receptor
TLR4	Toll-like receptor 4
TMEM119	Transmembrane protein 119
TNF	Tumor necrosis factor
TREM2	Triggering receptor expressed on myeloid cells 2
TREM2^R47H/+^	Triggering receptor expressed on myeloid cells 2 R4TH/+ variant
TREM2^T96K^	Triggering receptor expressed on myeloid cells 2 T96K variant
